# Effects of maternal western‐style diet on amniotic fluid volume and amnion VEGF profiles in a nonhuman primate model

**DOI:** 10.14814/phy2.13894

**Published:** 2018-10-23

**Authors:** Cecilia Y. Cheung, Victoria H. J. Roberts, Antonio E. Frias, Robert A. Brace

**Affiliations:** ^1^ Department of Obstetrics and Gynecology Oregon Health and Sciences University Portland Oregon; ^2^ Division of Reproductive and Developmental Sciences Oregon National Primate Research Center Portland Oregon

**Keywords:** Amniotic fluid index, high fat diet, macaque, soluble receptor, vascular endothelial growth factor

## Abstract

During pregnancy, high fat diet (HFD) induces maternal obesity, insulin resistance, and placental inflammatory responses that compromise placental and fetal development. Whether maternal HFD would adversely affect amniotic fluid volume (AFV) has not been explored. Vascular endothelial growth factor (VEGF) is expressed in the amnion and has been proposed as a regulator of AFV. Our aim was to investigate the effects of HFD on AFV and the associated changes in VEGF and soluble VEGF receptor 1 (sFlt‐1) expression profiles in three amnion regions of a nonhuman primate model. Further, we examined the relationships between VEGF expression and HFD‐induced changes in maternal metabolic status. Japanese macaques were maintained on control or HFD and amniotic fluid index (AFI) was measured as an ultrasonic estimate of AFV. Amniotic fluid VEGF concentrations were determined by ELISA and amnion VEGF and sFlt‐1 mRNA levels by real‐time RT‐qPCR. HFD increased maternal plasma triglyceride while glucose levels were unchanged. Maternal weight gain was found in diet‐sensitive animals whereas amniotic fluid VEGF concentration was reduced in diet‐resistant animals. HFD did not alter AFI and there was no correlation between AFI and maternal weight or amniotic fluid VEGF concentrations. VEGF mRNA levels were lowest in secondary placental amnion while sFlt‐1 mRNA were lowest in the primary placental amnion. HFD did not affect amnion VEGF or sFlt‐1 mRNA expression. These findings suggest that although maternal HFD increased maternal weight in diet‐sensitive and reduced amniotic fluid VEGF concentrations in diet‐resistant phenotype, AFV as indicated by the AFI, was not significantly affected.

## Introduction

Obesity is a leading cause of insulin resistance, cardiac abnormalities, diabetes, and metabolic syndrome. Although the causes are multifactorial, a diet high in caloric intake predisposes individuals to the development of obesity. Human epidemiological data demonstrate that, during pregnancy, diet‐induced maternal obesity is associated with adverse maternal conditions including hypertension and preterm delivery. Neonatal consequences include growth restriction and stillbirth (Frias and Grove 2012; Fisher et al. 2013; Schummers et al. 2015), hepatic and pancreatic inflammation (Grant et al. 2012; Nicol et al. 2013), and dysregulation of neuronal programming of energy balance (Sullivan et al. 2017). For the past decade, our group has utilized a Japanese macaque model of diet‐induced obesity to study the effects of high fat diet (HFD) during pregnancy on maternal, placental, fetal, and neonatal outcomes (McCurdy et al. 2009, 2016; Grayson et al. 2010; Nicol et al. 2013; Pound et al. 2014). In this model, pregnant animals chronically fed a HFD typically segregated into two phenotypes: HFD‐sensitive (HFD‐S) animals become obese and have dysregulation of insulin sensitivity, and HFD‐resistant (HFD‐R) animals remain lean and have normal insulin function (McCurdy et al. 2009; Frias et al. 2011). Maternal HFD with or without obesity has detrimental effects on placental blood flow, increases fetal body fat content and glycerol levels, and causes oxidative stress with cellular damage in fetal liver and skeletal muscle (McCurdy et al. 2009, 2016; Frias et al. 2011). In addition, we have shown that supplementation of a HFD with the anti‐inflammatory antioxidant resveratrol leads to improvement in maternal metabolic phenotype, uterine arterial volume blood flow, and placental function (Roberts et al. 2014).

During pregnancy, an amniotic fluid volume (AFV) in the normal range promotes the development of a healthy fetus. In contrast, AFVs outside of the normal range have the potential to adversely affect fetal growth as well as maternal and neonatal health. A reduced volume of amniotic fluid (oligohydramnios) can cause birth defects such as fetal pulmonary hypoplasia, premature delivery, umbilical cord compression, in utero fetal death, and an increased risk of cesarean section. Excess amniotic fluid (polyhydramnios) can lead to preterm delivery, premature rupture of fetal membranes, placental abruption, fetal malposition, and umbilical cord prolapse (Locatelli et al. 2004; Hamza et al. 2013; Morris et al. 2014). The regulation of AFV is determined primarily by the rate of intramembranous transport of amniotic fluid across the placental amnion from the amniotic compartment into fetal blood whereas minimal transfer occurs across the reflected amnion (Brace et al. 2014). The growth factor VEGF has been proposed to play an important role in the regulation of AFV by participating in regulating the rate at which vesicles transport fluid across the amnion (Cheung 2004; Brace and Cheung 2014; Sharshiner et al. 2017). In addition, VEGF gene‐expression in the amnion was shown to increase in conditions of increased intramembranous transport of amniotic fluid (Daneshmand et al. 2003; Cheung 2004). In nonpregnant adults, obesity is associated with elevated VEGF levels in the circulation and adipose tissue is the presumed source of the excess VEGF (Miyazawa‐Hoshimoto et al. 2003; Zafar et al. 2017). The high levels of VEGF, acting through its receptors, induce migration and proliferation of endothelial cells and increases vascular permeability to further expansion of adipose tissues. Thus, VEGF may play a role in the pathogenesis of obesity and metabolic disturbances.

Based on this assumption, obesity‐induced changes in amnion VEGF gene expression in pregnancy could modify AFV to potentially affect fetal and neonatal outcome. In an ovine model, maternal obesity was associated with reduced AFV (Satterfield et al. 2012). In pregnant sheep, diet‐induced maternal obesity is associated with decreased fetal placental arterial VEGF mRNA and protein expressions from mid‐gestation through term. This decline in angiogenic factor may function as a protective mechanism to reduce placental vascular development and thus maternal nutrient delivery to the fetus (Ma et al. 2010). In pregnant swine genetically predisposed to obesity, the number of conceptuses was found to be diminished and appeared to be related to lower placental expression of VEGF (Gonzalez‐Bulnes et al. 2012). These observations provide experimental evidence supporting the involvement of placental VEGF in HFD‐induced maternal obesity and poor pregnancy outcome. In humans, however, it is unclear whether maternal obesity due to HFD would lead to altered amniotic fluid dynamics and changes in amnion VEGF expression.

Of interest, resveratrol has been shown to suppress VEGF gene expression and protein levels in various cell types (Zhang et al. 2014; Kuroyanagi et al. 2015) including cancer cells (Tino et al. 2016; Kim et al. 2017). Although resveratrol supplementation could improve uterine artery volume blood flow in HFD animals (Roberts et al. 2014), it is unknown whether resveratrol would modulate AFV through alterations of VEGF levels in the amniotic compartment.

The present study was designed to investigate the effects of maternal high fat intake on AFV and VEGF expression in the amnion and to correlate these changes with maternal metabolic profile in order to determine whether HFD is a risk factor for AFV abnormalities. To determine amnion VEGF expression, we quantified VEGF concentrations in the amniotic fluid, analyzed the gene expression of VEGF in three regions of the amnion, and determined amnion gene expression of the antiangiogenic VEGF receptor soluble Flt‐1 (sFlt‐1) that specifically binds VEGF to reduce its bioavailability. Since the VEGF in amniotic fluid presumably is predominantly derived from the amnion, the concentration of amniotic fluid VEGF should reflect expression levels in the amnion. Further, because resveratrol has been proposed for therapeutic use to improve metabolic health (Shah et al. 2016; Menichini et al. 2018), we examined the effects of resveratrol on VEGF concentrations in amniotic fluid.

We utilized our Japanese macaque diet‐induced obesity model for these studies. Of note, nonhuman primates, similar to humans, have a hemochorial placenta, but the placenta is typically bi‐discoid with the umbilical cord attachment to the primary lobe and an accessory secondary lobe supplied by bridging vessels (Carter 2007). In humans, specific differences in gene expression profile exist in the amnion overlying the placenta as compared to the reflected amnion covering the membranous chorion (Han et al. 2008; Bednar et al. 2015). Whether differences exist in amnion that overlies the primary compared to secondary placenta in primates is unknown. In this study, we analyzed gene expression levels in the three regions of the amnion. Our objectives were to determine the effects of maternal HFD on (1) VEGF concentrations in amniotic fluid, (2) VEGF and sFlt‐1 gene expression profiles in the three (primary placental, secondary placental and reflected) amnion regions, and (3) relationships among VEGF expression, AFV and maternal metabolic status. Further, we explored whether resveratrol supplementation would mitigate the effects of HFD on VEGF expression and AFV status. We hypothesized that the diet‐induced increase in maternal weight is associated with a decrease in AFV and changes in amnion VEGF/sFlt‐1 gene expression, and that these effects could be ameliorated by resveratrol supplementation.

## Materials and Methods

### Animal preparation

All animal procedures were conducted in accordance with the guidelines of the Institutional Animal Care and Utilization Committee of the Oregon National Primate Research Center (ONPRC) and Oregon Health & Science University. The ONPRC abides by the Animal Welfare Act and Regulations of the USDA. Japanese macaques (Macaca fuscata) were maintained on a HFD (36% calories from fat; Purina Mills, Inc., St. Louis, MO, USA) supplemented with calorie‐dense treats for 4–7 years and throughout pregnancy, as previously described (Roberts et al. 2014; Harris et al. 2016). The composition of this diet represents a typical western style diet in regard to the saturated fat content. Control animals were fed standard chow (14% calories from fat) matched in micronutrient content. Resveratrol (high‐purity *trans*‐resveratrol, Resvida; DSM Nutritional Products, Inc., Parsippany, NJ, USA) was incorporated in the resveratrol‐supplemented HFD chow by the manufacturers (Purina Mills) at the time of production to a final concentration of 0.37%. In a subset of animals, resveratrol supplementation was initiated 3 months before the breeding season and continued throughout pregnancy. With this supplementation regiment, maternal fasting circulating resveratrol levels were ~1 ng/mL and postprandial levels were up to 70 ng/mL (Roberts et al. 2014). Animals were allowed to breed naturally, and pregnancies were identified by routine ultrasound. Gestational age dating was achieved by fetal biometry in early gestation.

Maternal metabolic parameters (body weight, plasma fasting glucose, triglycerides, insulin, and leptin) were assessed in the dams at regular intervals as described in detail previously (Roberts et al. 2014). Animals were subjected to an ultrasound exam at approximately 120 days gestation (0.7 term), as described in our recent publication (Cheung et al. 2018;). During the exam, the four‐quadrant amniotic fluid index (AFI) was measured (GE Voluson 730 Expert; Kretztechnik, Zipf, Austria) by one ultrasonographer (AEF) and used as an index of AFV. The AFI was utilized because it is noninvasive and is widely used in humans to provide a semi‐quantitative index of AFV. The HFD animals were subdivided into those that were sensitive to the HFD (HFD‐S, became obese and developed insulin resistance) and those that were resistant (HFD‐R, were neither obese nor insulin resistant) to the HFD. Amniotic fluid samples and samples of amniotic membranes (primary placental, secondary placental and reflected amnion) were collected at cesarean delivery at 130 days (0.75 term). The total number of pregnant macaques used for this study was 49 and were separated into control diet group (*n* = 20) and HFD group (*n* = 29). A subset of HFD animals (*n* = 9) were supplemented with resveratrol. Not all samples were available for collection from every animal because this study was one of the multiple studies that utilized this macaque preparation. The number of samples used for individual parameters are specified in the figure legends.

### VEGF ELISA

For quantification of VEGF in amniotic fluid, samples were centrifuged at 1800*g* for 15 min to remove particulates and analyzed for VEGF concentrations in triplicates by solid phase sandwich ELISA (Human VEGF Quantikine ELISA kit, R & D Systems) using procedures provided by the manufacturer. This assay was specifically designed to recognize VEGF_165_, the most abundantly expressed isoform, with >50% cross‐reactivity with VEGF_121_ and VEGF_189_. The intraassay and interassay coefficients of variance were 4.5% and 7%, respectively. Sensitivity of the assay for VEGF was 9 pg/mL and specificity >85%. The cross‐reactivity for VEGF‐B, VEGF‐C, VEGF‐D, PlGF, and related angiogenic factors was <0.5%.

### RT‐qPCR

The VEGF and sFlt‐1 mRNA levels were determined by real time RT‐qPCR as previously described (Cheung et al. 2018). Total RNA (2 *μ*g) was reversed transcribed using MultiScribe reverse transcriptase. Sample cDNA (25 ng) was amplified in triplicate using a *TaqMan* Gene Expression Assay system (Applied Biosystems, Thermo Fisher Scientific) and custom designed (Primer Express^®^ Software v3.0. Applied Biosystems, Thermo Fisher Scientific) *Macaca Mulatta*‐specific primers for VEGF (forward: 5′‐GAGCGGAGAAAGCATTTGTTTGTA‐3′, and reverse: 5′‐GCAACGCGAGTCTGTGTTTT‐3′); and for sFlt‐1 (forward: CCTCCAGAAGAAAGAAGTTACAATCA‐3′, and reverse: 5′‐ TTTGGAGATCCGAGAGAAAACAG‐3′). The primers for VEGF were designed to amplify the three major isoforms of VEGF‐A: VEGF_121_, VEGF_165_, and VEGF_189_. The temperature profile was: initial two‐step hold at 50°C for 2 min and 95°C for 10 min, followed by 40 cycles of 15 sec at 95°C and 1 min at 60°C. Two endogenous references, 18S ribosomal RNA and PES1 (pescadillo ribosomal biogenesis factor 1), were used as house‐keeping genes. These two constitutively expressed genes were selected as internal references because of their stable expression profile under various experimental conditions including high fat intake. A standard curve was incorporated for VEGF, sFlt‐1 and each of the two endogenous references. The relative quantities of VEGF and sFlt‐1 mRNA were determined by the comparative CT method after normalization to the geometric mean of the CT values for the two house‐keeping genes (18s and PES1). CT values of the house keeping genes and their geometric means were not altered by diet. For comparison of expression levels, fold change in VEGF and sFlt‐1 mRNA was calculated as the 2^−∆∆C^_T_ value using the control diet group as the calibrator.

### Statistical analysis

Data are presented as mean ± SE. Parametric one‐factor analyses of variance (one‐factor ANOVA) and t‐tests were used for group comparisons. If the null hypothesis was rejected by the ANOVA, Fisher's least significant difference was used for post hoc testing. Relationships between AFI, amniotic fluid VEGF concentrations, and amnion VEGF or sFlt‐1 mRNA levels were determined by least squares bivariate regression. Pearson's correlation coefficient (r) was used to express the degree of linearity between two variables. Data were logarithmically transformed as needed to normalize variances prior to analysis; transformed data are plotted on log scales in the figures. *P* ≤ 0.05 was considered significant.

## Results

### Effects of diet on maternal weight and amniotic fluid index

Maternal weight at 130 days gestation (0.75 term) was 10.27 ± 0.43 kg in the control dams. High fat diet significantly increased maternal weight to 12.81 ± 0.56 kg (*P* < 0.05) and this was due to the weight gain in the sensitive but not in resistant animals (Table 1).

**Table 1:** Maternal weight and plasma concentrations as functions of diet at 130 days gestation (mean ± SE)

	Control diet	High fat diet combined	High fat diet resistant	High fat diet sensitive
Weight (kg)	10.27 ± 0.43 (*n* = 20)	12.81 ± 0.56^a^ (*n* = 29)	11.35 ± 0.67 (*n* = 13)	13.99 ± 0.75^b^ (*n* = 16)
Glucose (mg/mL)	45.3 ± 2.6 (*n* = 19)	47.5 ± 2.7 (*n* = 22)	43.7 ± 3.9 (*n* = 9)	50.2 ± 3.7 (*n* = 13)
Triglycerides (mg/mL)	0.376 ± 0.057 (*n* = 13)	0.565 ± 0.055^a^ (*n* = 18)	0.612 ± 0.69 (*n* = 8)	0.527 ± 0.083 (*n* = 10)
Insulin (mU/mL)	22.2 ± 5.3 (*n* = 20)	45.0 ± 10.4 (*n* = 29)	28.3 ± 4.9 (*n* = 13)	58.5 ± 17.9^b^ (*n* = 16)
Leptin (ng/mL)	55.3 ± 8.1 (*n* = 17)	74.3 ± 6.3 (*n* = 26)	71.3 ± 10.9 (*n* = 12)	76.8 ± 7.4 (*n* = 14)

a *P < 0.05 compared to control diet by *t*‐test.

b *P* < 0.05 compared to control diet by ANOVA post hoc testing.

**Figure 1:**
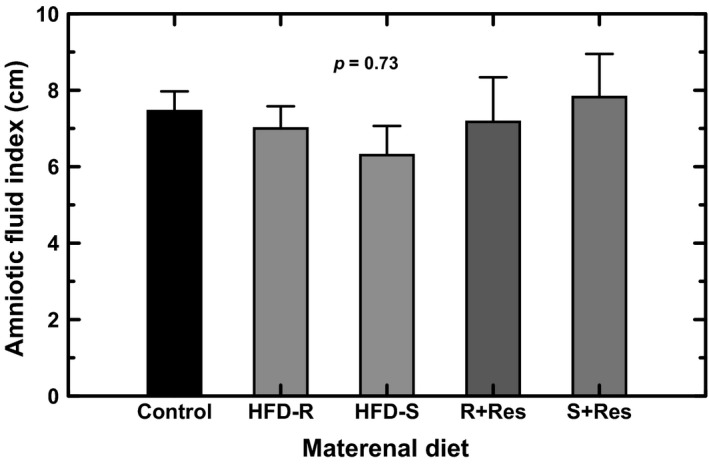
Amniotic fluid index (AFI) in Japanese macaques under control (*n* = 11), high fat diet (HFD) resistant (HFD‐R, *n* = 6) and HFD sensitive (HFD‐S, *n* = 7) conditions. Subsets of HFD resistant (R+resv, *n* = 5) and HFD sensitive (S+resv, *n* = 4) animals were fed a diet supplemented with resveratrol. The AFIs in HFD‐S and HFD‐R animals as well as those fed resveratrol supplementation were not significantly different from that in the control group (one‐factor ANOVA,* P* = 0.73).

The AFI measured in the control animals (*n* = 11) averaged 7.45 ± 0.52 cm and in HFD (*n* = 13) animals was 6.63 ± 0.48 cm. The AFI in HFD group was statistically not different (*P* = 0.26) from that in the control group. Further, there was no relationship between AFI and maternal weight in combined control and HFD animals (*r* = 0.16, *P* = 0.39). When the HFD animals were separated into resistant and sensitive subgroups, similarly there was no difference in AFI among the subgroups or when compared to the control (Fig. 1). Resveratrol supplementation did not modify the AFI in HFD‐R and HFD‐S animals (Fig. 1).

The metabolic status of the animals is shown in Table 1. Compared to the control group, maternal triglycerides levels were significantly higher in HFD groups (resistant and sensitive groups combined). Maternal insulin levels were elevated (*P* < 0.05) in the HFD‐S group but not in the HFD‐R group compared to control while glucose levels in the control and both HFD groups were not different. Plasma leptin levels were similar among the control and both HFD groups. In the subset of resveratrol supplemented animals (*n* = 9, data not shown), resveratrol did not affect maternal weight, insulin concentration, leptin concentration, or glucose concentration. However, resveratrol blocked the rise in maternal triglycerides in both the HFD‐R (*n* = 5) and HFD‐S (*n* = 4) animals (one‐factor ANOVA, *P* = 0.036).

### Effects of diet on amniotic fluid VEGF Concentrations

**Figure 2:**
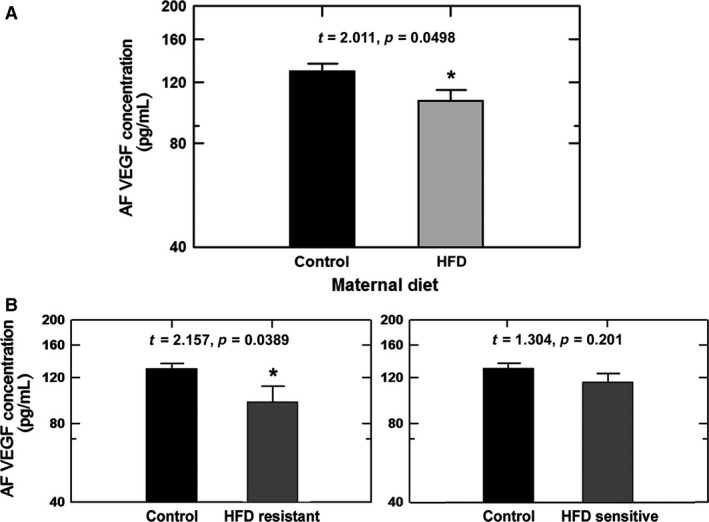
Effects of maternal diet on amniotic fluid (AF) VEGF concentrations in Japanese macaques. (A) AF VEGF concentrations in high fat diet animals (HFD,* n* = 29) were lower than control diet animals (*n* = 20) (one‐factor ANOVA,* P* = 0.05). (B) AF VEGF concentrations in HFD animals subdivided into resistant (*n* = 13, one‐factor ANOVA,* P* = 0.039 compared to control) and sensitive (*n* = 16, one‐factor ANOVA,* P* = 0.20 compared to control) groups. *,* P* < 0.05 compared to control group by *t*‐test.

**Figure 3:**
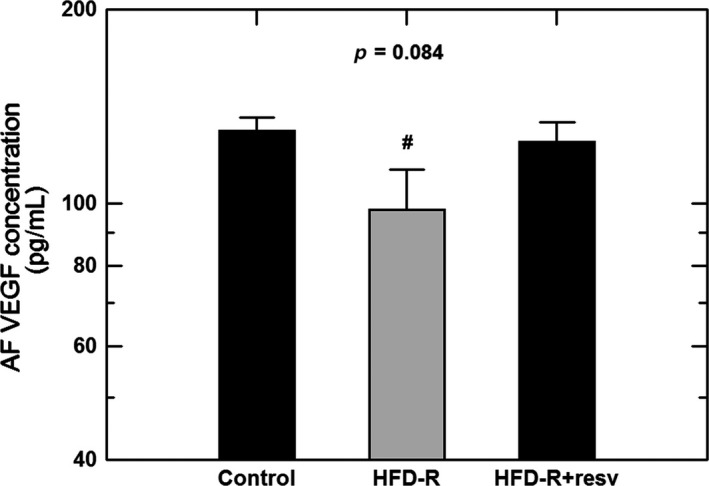
Comparison of amniotic fluid (AF) VEGF concentrations in control (*n* = 20) and high fat diet resistant macaques (HFD‐R, *n* = 13). A subgroup of resistant animals were fed HFD supplemented with resveratrol (HFD‐R+resv, *n* = 5). VEGF concentrations in the HFD‐R animals treated with resveratrol were significantly higher than that in HFD‐R animals without supplementation but not different from the control group (one‐factor ANOVA,* P* = 0.084). ^**#**^, *P* < 0.05 compared to control group.

In the cohort of Japanese macaques studied, amniotic fluid VEGF concentrations were not different between the male (126 ± 7 pg/mL) and female (122 ± 8 pg/mL) fetuses (*P* = 0.74) for control and HFD combined. Maternal HFD reduced mean levels of VEGF in amniotic fluid and the reduction was significant when both genders were combined (*P* < 0.05, Fig. 2A). When the HFD group was sub‐divided by diet sensitivity, the decrease in VEGF concentration was observed only in the HFD‐R group (*P* < 0.05) but not in the HFD‐S group (Fig. 2B). In a subgroup of HFD‐R, animals fed a resveratrol supplemented diet, the amniotic fluid VEGF concentrations were no longer different from those in the control diet group (Fig. 3).

### Effects of diet on amnion VEGF and sFlt‐1 gene expression

**Figure 4:**
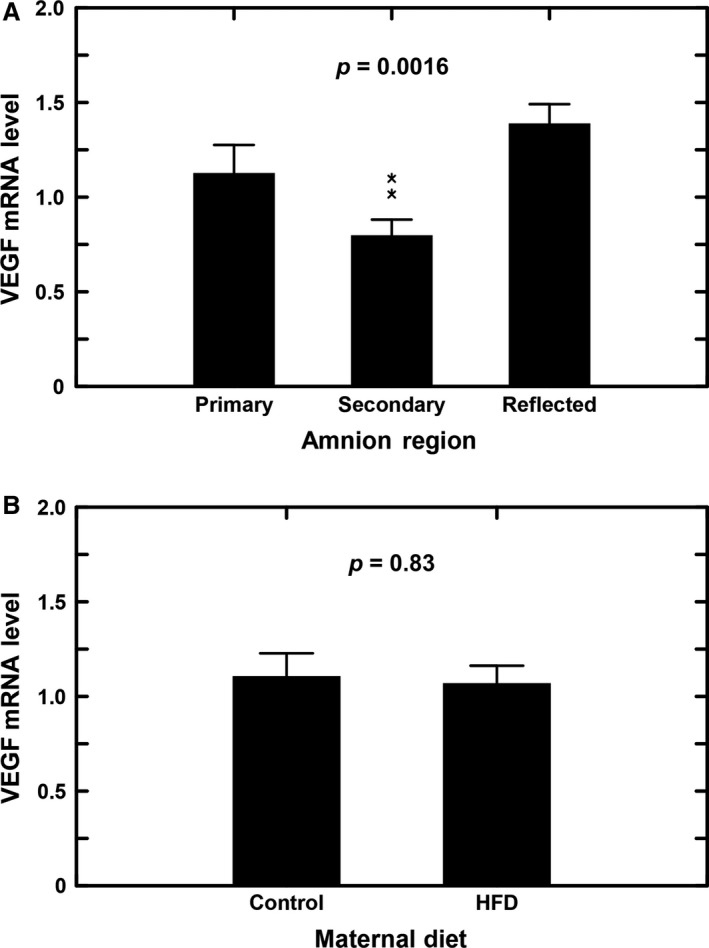
Effects of maternal diet on VEGF mRNA expression in three amnion regions of Japanese macaques. (A) Regional differences in VEGF mRNA levels of control and high fat diet (HFD) groups combined. VEGF mRNA levels in secondary placental amnion (*n* = 8) were significantly lower than that in primary placental amnion (*n* = 12) and reflected amnion (*n* = 14) (one‐factor ANOVA,* P* = 0.0016). (B) VEGF mRNA levels in the amnion (combined primary placental, secondary placental and reflected amnion) of the control animals (*n* = 13) were not different from that in HFD animals (*n* = 21. one‐factor ANOVA,* P* = 0.83). **, *P* < 0.01 compared to primary placental and reflected amnion.

**Figure 5:**
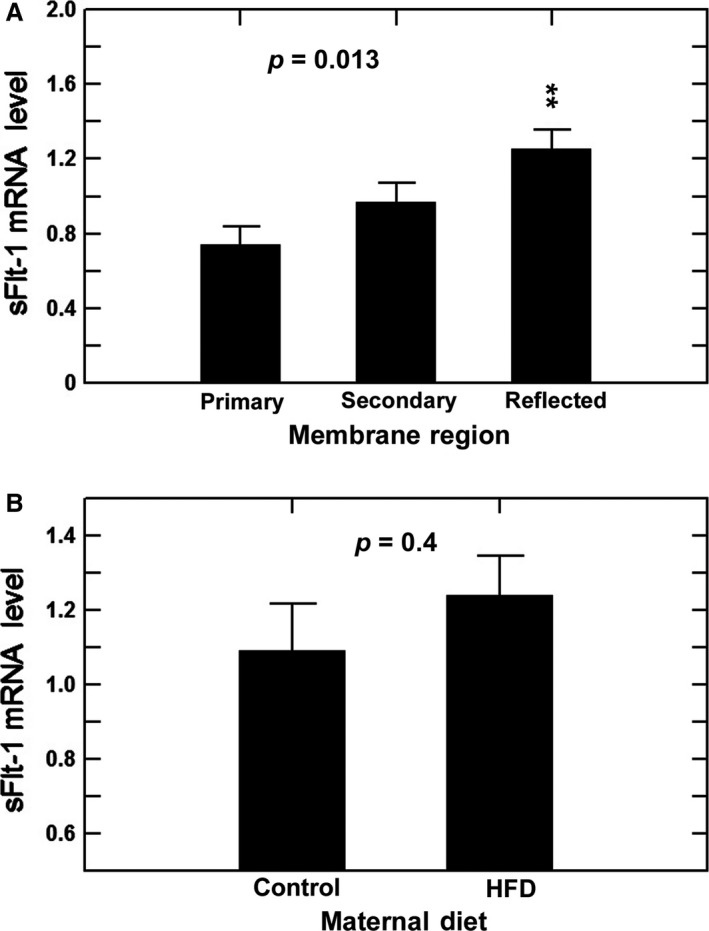
Effects of maternal diet on sFt‐1 mRNA expression in the three amnion regions of Japanese macaques. (A) Regional differences in sFlt‐1 mRNA levels of control and high fat diet (HFD) groups combined. sFlt‐1 mRNA levels in reflected amnion (*n* = 13) were significantly higher than that in primary placental amnion (*n* = 12) but not different from the secondary placental amnion (*n* = 8) (one‐factor ANOVA,* P* = 0.013). (B) sFlt‐1 mRNA levels in the amnion (combined primary placental, secondary placental and reflected amnion) of the control animals (*n* = 12) were not different from that in HFD animals (*n* = 21, one‐factor ANOVA,* P* = 0.40). **,* P* < 0.01 compared to primary placental amnion.

The gene expression of VEGF was analyzed in three regions of the macaque amnion. In control animals, VEGF mRNA levels in the secondary placental amnion were significantly lower than those in the primary placental and reflected amnion (*P* < 0.05, Fig. 4A). Levels in the reflected amnion were modestly (nonsignificantly) higher as compared to the primary placental amnion. There were no differences in VEGF mRNA levels between control and HFD animals for all three amnion regions analyzed individually or when the regions were combined (Fig. 4B). When the HFD animals were subdivided into sensitive and resistant subgroups, again there were no significant regional differences in amnion VEGF mRNA levels between control and the two HFD groups (one factor ANOVA, *P* = 0.99). Further, there were no diet‐induced gender differences in VEGF mRNA levels within the three amnion regions and when both diets were combined (one‐factor ANOVA, *P* = 0.65). Regional differences in amnion sFlt‐1 mRNA levels were found in that levels in the reflected amnion were higher than in the primary but not secondary placental amnion (Fig. 5A) for the control, HFD‐R, and HFD‐S animals combined. When separated into control and HFD groups, no differences in sFlt‐1 levels were noted in the combined amnion regions (Fig. 5B).

### Relationships among amnion VEGF expression, AFV and maternal metabolic status

**Figure 6:**
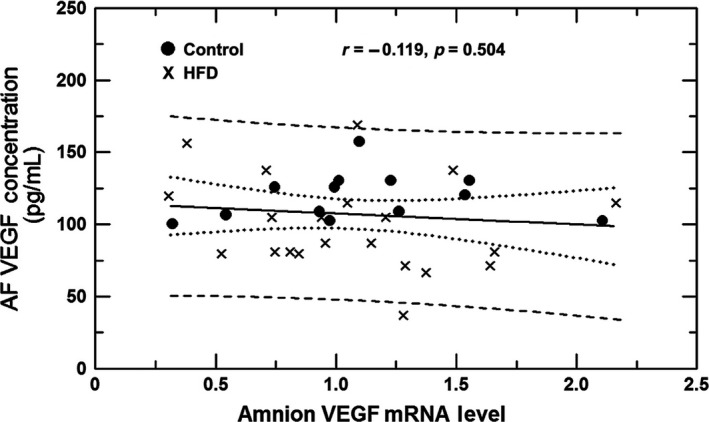
Regression analysis between amniotic fluid (AF) VEGF concentration and amnion VEGF mRNA level in control and high fat diet (HFD) animals. The analysis was performed on mRNA levels of combined amnion regions (primary placental, secondary placental, and reflected amnion). The relationship between AF VEGF concentration and amnion mRNA level was not significantly different (bi‐variate regression, *r* = −0.119, *P* = 0.50).

To determine whether amniotic fluid VEGF concentrations and amnion VEGF or sFlt‐1 mRNA levels were correlated, bivariate linear regression was performed using values from the three amnion regions combined. No significant relationship was found between amniotic fluid VEGF concentrations and amnion VEGF mRNA (Fig. 6) or amnion sFlt‐1 mRNA levels (*P* = 0.60). In addition, amnion VEGF mRNA was not correlated with amnion sFlt‐1 mRNA levels (*P* = 0.20).

**Figure 7:**
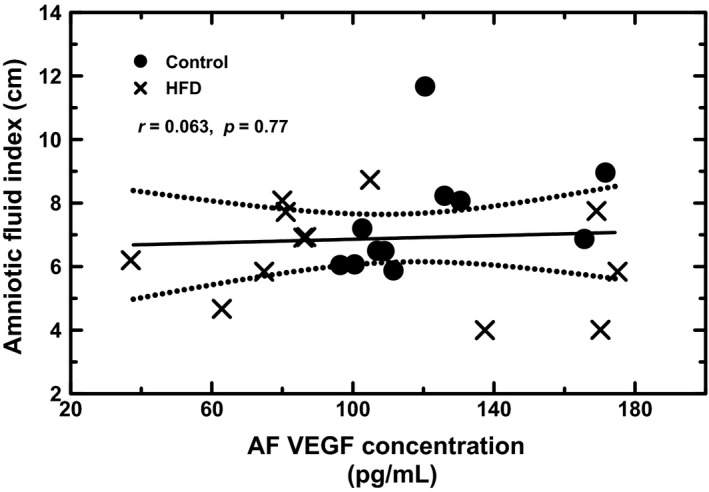
Regression analysis between amniotic fluid index (AFI) and amniotic fluid (AF) VEGF concentration in control and high fat diet (HFD) macaques. There was no statistically significant relationship between AFI and AF VEGF concentrations irrespective of maternal diet (bi‐variate regression, *r* = 0.063, *P* = 0.77).

There was no correlation between maternal weight and amniotic fluid VEGF concentrations (*P* = 0.20) or amnion VEGF mRNA levels (*P* = 0.24) in the combined control and HFD animals, irrespective of insulin sensitivity in the HFD groups. When the HFD‐R and HFD‐S groups were analyzed separately, again there was no correlation between maternal weight and amniotic fluid VEGF concentration or amnion VEGF mRNA in either groups. In addition, the AFI did not correlate with amniotic fluid VEGF concentrations (*P* = 0.52, Fig. 7), amnion VEGF mRNA (*P* = 0.72), or amnion sFlt‐1 mRNA levels (*P* = 0.48), irrespective of diet or insulin sensitivity.

## Discussion

The present study demonstrated that, in Japanese macaques, HFD increased maternal weight and plasma triglyceride levels as previously reported (McCurdy et al. 2009; Frias et al. 2011). Further analysis showed that obesity developed only in a subgroup of animals sensitive to the HFD and this was associated with elevated insulin levels. The HFD resistant animals remained lean with normal insulin, consistent with our published findings in studies using this model (McCurdy et al. 2009; Frias et al. 2011). This suggests that the diet‐induced increase in insulin levels may predispose the individual to obesity.

During pregnancy, a normal volume of amniotic fluid is essential for optimal fetal growth and development. In pregnant sheep, maternal obesity was found to be associated with reduced AFV (Satterfield et al. 2012). In contrast, our present findings in pregnant macaques indicate that high fat intake with or without maternal weight increase had no significant effects on AFV, since AFIs were comparable in animals that became obese and those that remained lean. These results do not support our initial hypothesis that diet‐induced maternal weight gain would be associated with changes in amniotic fluid status.

Amniotic fluid VEGF concentrations decreased in HFD animals and this was not related to changes in amnion VEGF gene expression. The reduction in amniotic fluid VEGF was found only in the HFD‐R group that neither became obese nor developed insulin resistance. This decrease was not observed in the HFD‐S animals that gained weight with elevated plasma insulin levels. These results in macaques differ from those in nonpregnant human adults where obesity is associated with increases in circulating VEGF levels (Miyazawa‐Hoshimoto et al. 2003; Zafar et al. 2017). Such differences could be due to amniotic fluid versus circulating concentrations or to species variations. In the HFD‐R animals, the reduced amniotic fluid VEGF concentration was not related to maternal obesity or AFV since there was no correlation between amniotic fluid VEGF concentrations and either maternal weight or AFI. This reduction in VEGF concentration most likely reflects an amniotic response to the diet in the resistant phenotype to potentially minimize the impact of the increased fat intake. Of note, our studies showed that supplementation of maternal diet with resveratrol reversed the effect of HFD on amniotic fluid VEGF concentrations in HFD‐R animals even though resveratrol did not affect AFI in both control and HFD (sensitive and resistant) animals. We have previously shown that resveratrol use during pregnancy was beneficial to maternal and placental metabolic parameters, however, fetal pancreatic development was compromised (Roberts et al. 2014).

In pregnant sheep, diet‐induced obesity was associated with decreased fetal placental arterial VEGF gene expression (Ma et al. 2010). The authors suggested that this decline may function as a protective mechanism to reduce placental vascular development. In the present study, we did not observe changes in amnion VEGF gene expression in response to the HFD. VEGF mRNA levels in three regions of the macaque amnion were not altered by the HFD, irrespective of whether the animals gained weight or remained lean. Further, the VEGF mRNA profiles in the three regions of the amnion were not affected by maternal diet status, weight change, or insulin sensitivity. High fat diet similarly did not modify sFlt‐1 mRNA levels in the three amnion regions and there was no relationship between amnion sFlt‐1 levels and amnion VEGF mRNA or amniotic fluid VEGF concentrations. This lack of relationship suggests that the abundance of sFlt‐1 in amnion tissue does not modulate VEGF expression in the amnion or VEGF concentrations in the amniotic fluid. The absence of HFD effects on amnion VEGF and sFlT‐1 gene expression are similar to findings in our recent study of pregnant macaques fed a HFD (Cheung et al. 2018) in that expression levels of five aquaporins in the amnion was not affected by the high fat intake in animals with the resistant phenotype. Of interest, analysis of regional differences in amnion gene expression demonstrated that VEGF and sFlt‐1 levels differed among the three regions with VEGF expression lowest in secondary placental amnion and highest in reflected amnion, while sFlt‐1 expression lowest in the primary placental amnion but highest in the reflected amnion. These regional differences were consistent with the differential gene expression profiles characteristic of different amnion regions (Han et al. 2008), as well as with the differential aquaporin gene expression shown in our recent HFD pregnant macaque (Cheung et al. 2018) and human (Bednar et al. 2015) studies. The significance of such differences is unclear and may be related to the permeability characteristics of the three amnion regions.

In conclusion, based on the findings in a nonhuman primate model of HFD, a diet high in fat during pregnancy could exert differential effects on the amnion that are dependent on maternal sensitivity to the diet. In the sensitive phenotype, HFD leads to maternal obesity and increased plasma insulin levels without significant effects on AFV, amniotic fluid VEGF concentrations and amnion VEGF, and sFlt‐1 gene expression. Conversely, in the diet resistant phenotype, obesity does not develop and insulin sensitivity is normal. An unexpected finding was the reduced amniotic fluid VEGF concentrations in the resistant animals without changes in AFI or amnion VEGF and sFlt‐1 expressions. Based on these findings, we speculate that there may be an association between reduction in amniotic fluid VEGF levels and development of resistance to certain adverse effects of HFD. The resistant phenotype may, in part, be reflective of reduced VEGF mediated fetal‐placental angiogenesis to minimize nutrient transfer.

## Conflicts of Interest

The authors declare that there are no conflicts of interest, and there are no disclosures to declare.
